# One year after first case of COVID-19 in Ghana: epidemiology, challenges and accomplishments

**DOI:** 10.11604/pamj.2021.39.226.29069

**Published:** 2021-08-04

**Authors:** Seth Kwabena Amponsah, Benjamin Tagoe, Daniel Kwame Afriyie

**Affiliations:** 1 Department of Medical Pharmacology, University of Ghana Medical School, Accra, Ghana,; 2 Fulfilment Operations and Academy, Zipline Ghana, Accra, Ghana,; 3 Ghana Police Hospital, Accra, Ghana

**Keywords:** Accomplishments, challenges, COVID-19, Ghana, vaccine

## Abstract

Coronavirus disease 2019 (COVID-19) caused by the novel coronavirus, has affected many lives, health systems and economies across the globe. Countries in both resource-rich and poor have equally been affected. In Ghana, COVID-19 has caused morbidity and mortality among the populace. The first two cases of COVID-19 were reported in Ghana in March 2020. At the onset of the pandemic in Ghana, there were challenges in securing isolation centers and quarantine facilities. Nonetheless, the government of Ghana put in place a number of measures in line with World Health Organization (WHO) guidelines, to halt the spread of the virus. Some measures taken by the government included partial lockdown of areas deemed hotspots for the spread of the virus. In April 2020, Ghana was ranked number one among African countries in administering tests per million people, because of the effective “trace and test” approach. The government of Ghana also encouraged local manufacturing of personal protective equipment, antivirals and hand sanitizers to help meet the demand of the nation. There were also restrictions on public gathering within the early parts of 2020, and these were eased with time. In February 2021, Ghana became the first country to receive vaccines through the COVAX initiative with a delivery of 600,000 doses of Oxford-AstraZeneca vaccines. The efforts by Ghana to deal with the COVID-19 pandemic have been commendable. Not withstanding, the adverse impact of the COVID-19 on public health in Ghana has been significant, and there is still a lot to learn from other countries in the sub-region, and the world as whole.

## Perspective

Coronavirus disease 2019 (COVID-19) caused by severe acute respiratory syndrome coronavirus 2 (SARS-CoV-2), has affected almost all countries worldwide. The negative impact of COVID-19 on countries cannot be overemphasized [1]. The first case of COVID-19 was in the city of Wuhan in China in December, 2019. Patients initially showed normal clinical symptoms of respiratory infection while on admission, until a rapid transformation predisposed them to an acute respiratory syndrome. In July 2020, the Word Health Organization (WHO) reported that COVID-19 case fatality ratio worldwide was 4.91% based on the 10,357,652 confirmed cases and 508,055 deaths [2]. As at 27^th^ March 2021, the WHO reported that there had been 125,781,957 confirmed cases of COVID-19, and 2,759,432 deaths, suggesting a case fatality ratio of 2.2% [3]. There were initial projections that COVID-19 will pose a significant challenge to health systems in Africa due to existing high prevalence rates of other infectious diseases and non-communicable diseases, compounded by high rates of antimicrobial resistance alongside a disproportionate burden of poverty. It was also anticipated that COVID-19 would further compound challenges with finances, human and healthcare resources including intensive care unit beds [4]. However, the African region has recorded relatively lower numbers of severe COVID-19 cases and case fatality rates. Also, the proportion of patients requiring intensive care management in Africa is relatively low. Most patients recover asymptomatically and are discharged. Generally, a low proportion require admission at treatment centers. Not withstanding, the negative impact of COVID-19 on public health systems in Africa remains significant [5].

**Figure 1:**
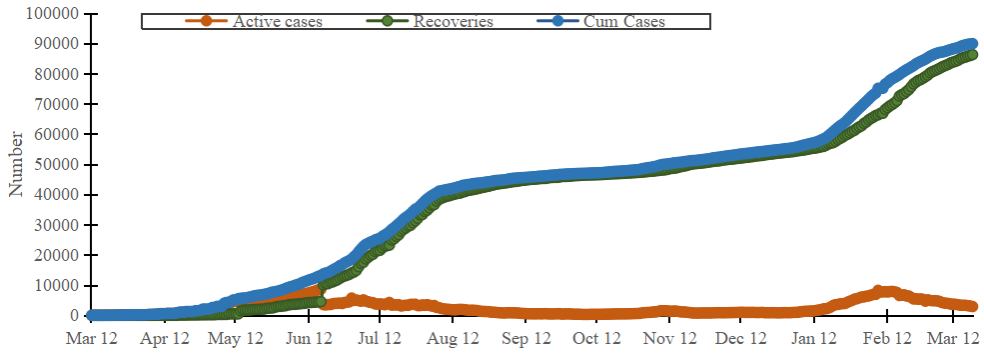
cumulative COVID-19 cases in Ghana, March 2020-March 2021. Source: Ghana Health Service

Ghana recorded its first two cases of COVID-19 on 12^th^ March 2020. In the first quarter (March 2020 to May 2020) of the pandemic in Ghana, the cumulative COVID-19 cases were less than 10,000 [6]. The numbers recorded in Ghana were almost same or less in some other African countries. In the second quarter (June 2020 to August 2020), there was a sharp increase in the cumulative number of COVID-19 cases [7]. However, the proportion of individuals that recovered or were discharged from treatment centers was remarkable. In the last quarter (December 2020 to February 2021), there was a second wave that resulted in a significant increase in number of COVID-19 cases, with more deaths (Figure 1). As at 13^th^ July 2021, the cumulative number of COVID-19 cases in Ghana was 97,585. There had been 801 deaths and 94,537 recoveries [8]. Ghana, like many African countries, has had challenges with dealing with both communicable and non-communicable diseases, in the midst of the COVID-19 pandemic. In addition, febrile conditions such as malaria, urinary tract infection and typhoid fever complicate timely detection of COVID-19 [9]. Furthermore, access to clean water has been a significant challenge to millions of inhabitants in Ghana, thus, a major limitation to proper and effective hand washing [10]. At the onset of the COVID-19 pandemic in Ghana, there were challenges in securing isolations centers and quarantine facilities. The government of Ghana, therefore, took measures such as renting hotels, churches and setting up temporary structures. Some of these were used for mandatory 14-day quarantine of travelers arriving into the country [11]. The government of Ghana amongst its plans to reduce spread of SARS-CoV-2 instigated mass testing among citizens, however, there were limited number of testing centers. Low physician-to-patient ratio, lack of intensive care unit (ICU) beds and ventilators were some other challenges to healthcare stakeholders in Ghana [12]. There were other health concerns associated with the pandemic in Ghana, one of which was an outbreak of meningitis in the Upper West region that led to 33 deaths in April 2020. There was the notion that this did not receive same attention as COVID-19 [13]. Routine follow-up of patients with non-communicable disease such as hypertension also declined during COVID-19 pandemic in Ghana. Generally, public healthcare systems focused on COVID-19, with a shift in funding to the prevention and management COVID-19, to the detriment of other diseases [14]. Furthermore, lack of extensive education about COVID-19 made some locals have the notion that COVID-19 was unreal. Some researchers have linked the comparatively low COVID-19 confirmed cases and deaths in Africa to under-reporting, reliability of testing equipment, and inadequate testing of the masses [15]. In Ghana, there were only 3 public testing centers (Noguchi Memorial Institute for Medical Research, Kumasi Centre for Collaborative Research and the National Public Health Reference Laboratory) at the start of the outbreak. However, there were systems in place to transport samples of suspected cases to these public testing centers. By June 2020, the number of testing centers had increased to 10 [16]. COVID-19 also negatively affected Ghana´s economy. Large segments of the population lost income due to lay-offs. The government of Ghana was unable to provide enough financial compensation to sustain households and this was a major problem especially during partial lockdown from 30^th^ March to 19^th^ April 2020 [17].

During the early stages of COVID-19 pandemic in Ghana, the government of Ghana instigated a partial lockdown in some hotspot areas. The partial lockdown was to, amongst others, limit the spread of the virus, provide adequate care for the sick, and enhance contact tracing and testing [18]. Following the partial lockdown, Ghana, like many other developed countries, implemented “mitigation-only” measures that included; testing-tracing-isolation of suspected cases, compulsory wearing of facial masks in public places, and banning of mass gathering [19]. Stringent measures were also put in place to ensure good hygienic practices at public places such as restaurants and bars. As a result of the inability to import sufficient equipment due to lockdown in most exporting nations, the Government of Ghana also introduced measures to facilitate increased local production of personal protective equipment, antivirals and hand sanitizers to help meet the demands of the nation [18]. In April 2020, scientists at the University of Ghana successfully sequenced genomes of SARS-CoV-2, obtaining important information about the genetic composition of viral strains in 15 of the confirmed cases in Ghana [20]. In July 2020, Ghana Infectious Disease Centre (GIDC) was also opened to improve medical diagnostic and research capacity of Ghana with regard to infectious diseases [21]. Furthermore, the government of Ghana announced some relief packages to ameliorate the hardship brought onto Ghanaians as a result of the COVID-19 pandemic. Some of these included free water from the period of April 2020 to December 2020. This was an incentive to help promote personal hygiene (hand washing). There were also incentives for frontline health workers, which included exemption from payment of tax on their employment emoluments, a daily allowance of GHS150 (approximately US$26) payable to those undertaking contact tracing, and additional allowance of 50% of their basic salary per month. Additionally, the frontline workers were to get an insurance package, with an assured amount of GHS350,000 (approximately US$60,345) [22].

Through the Ministry of Health in Ghana, the populace was educated on the need to practice social distancing and wear face masks. Citizens were urged to frequently wash their hands with soap under running water and apply alcohol-based hand rubbing lotion after drying hands [23]. The various stakeholders of health in the country contributed their quota in ensuring that there was nationwide surveillance, mass testing and case isolation to prevent resurgence. Banks, pharmacies, and grocery shops also provided hand sanitizers, spray disinfectants, veronica buckets (with water and soap) and disposable paper towels at entry points. The use of contactless payments methods such as mobile banking services was encouraged. In Ghana, the Ministry of Health recommended a combination of hydroxychloroquine with azithromycin or doxycycline in the management of mild to moderate cases of COVID-19. The aforementioned drugs are combined with convalescent plasma and methylprednisolone to manage severe cases without acute respiratory distress syndrome. If there is evidence of acute respiratory distress syndrome in a patient, remdesivir and tocilizumab are added to standard regimens. These pharmacological approaches appear to have helped reduce morbidity and mortality associated with COVID-19 [24]. On 24^th^ February 2021, Ghana became the first country to receive delivery of 600,000 doses of Oxford-AstraZeneca COVID-19 vaccines through the COVAX initiative [25]. Ghana began its coronavirus vaccination on 2^nd^ March 2021. The first phase of vaccinations prioritized frontline health workers and other populace who were at high risk of contracting and showing severe forms of the COVID-19. Also, during this first phase, some prominent public officials including the president and vice-president, with their spouses, received the COVID-19 vaccine. This was to boost public confidence ahead of mass vaccination [26].

As at 26^th^ March 2021, nearly 491,022 persons had received the first dose of the Oxford-AstraZeneca COVID-19 vaccine [27]. The populace vaccinated include front-line health workers, adults aged 60 years and above, and people with underlining health conditions. The others were frontline security personnel, frontline government officials, and all frontline workers in the formal sector. Data suggested that there were about 1000 reports of adverse drug reactions received by the Food and Drugs Authority as at 16^th^ March 2021 [28]. Some of these adverse reactions reported included fever, sweating, headache, weakness, chills and bodily pains. On 19^th^ May 2021, Ghana commenced phase two of its mass vaccination against COVID-19. About 350,000 doses of Oxford-AstraZeneca vaccines delivered to Ghana through the COVAX facility were expected to have been administered by health officials. Scores of persons who took their first doses about two months ago received their second dose. Despite the aforementioned, the Ministry of Health (Ghana) has had failed attempts to procure Sputnik V vaccines directly from Russian authorities and individuals, amid global scarcity. As at 10^th^ June 2021, the World Bank approved a US$200 million Ghana COVID-19 Emergency preparedness and response project second additional financing. This was to support the Government of Ghana to procure and deploy COVID-19 vaccines for 13 million people in Ghana. Thus far, Ghana has done remarkably well in containing SAR-CoV-2. Nonetheless, we propose solutions to deal with current COVID-19 challenges in Ghana (Table 1). As the COVID-19 pandemic continues across the world, public health officials are watching certain mutations and variants of the SARS-CoV-2 that may be more contagious or deadly than the original strain. The Delta variant of SARS-CoV-2 is of concern to the WHO because it is known to have increased transmissibility. This has been demonstrated in several countries. The differences in prevalence and mortality rates across countries may suggest that variables such as age and ethnicity could be predictors of COVID-19 severity, as such country-specific approaches may be required to effectively control the spread of SAR-CoV-2. Furthermore, continuous research should be conducted to assess efficacy of COVID-19 vaccines in various populations.

**Table 1:** current challenges in the fight against COVID-19 in Ghana and proposed solutions

Challenges	Solutions
Community infection of COVID-19 Delta variant	Nationwide surveillance, mass testing and case isolation
Few COVID-19 testing centers	Setting up of at least one COVID-19 testing center in each region of Ghana
Lax among populace in following COVID-19 protocols	Continuous education on COVID-19 protocols and the need to abide by them. Responsibility and accountability of systems and societies.
Vaccination of rest of populace	Mass vaccination through global collaboration
